# Evaluation of complexity and deliverability of IMRT treatment plans for breast cancer

**DOI:** 10.1038/s41598-023-48331-x

**Published:** 2023-12-06

**Authors:** Longyan Duan, Weixiang Qi, Yi Chen, Lu Cao, Jiayi Chen, Yibin Zhang, Cheng Xu

**Affiliations:** Department of Radiation Oncology, Ruijin Hospital, Shanghai Jiao Tong University School of Medicine, Shanghai, 200025 China

**Keywords:** Breast cancer, Applied physics

## Abstract

This study aimed to predict the outcome of patient specific quality assurance (PSQA) in IMRT for breast cancer using complexity metrics, such as MU factor, MAD, CAS, MCS. Several breast cancer plans were considered, including LBCS, RBCS, LBCM, RBCM, left breast, right breast and the whole breast for both Edge and TrueBeam LINACS. Dose verification was completed by Portal Dosimetry (PD). The receiver operating characteristic (ROC) curve was employed to determine whether the treatment plans pass or failed. The area under the curve (AUC) was used to assess the classification performance. The correlation of PSQA and complexity metrics was examined using Spearman’s rank correlation coefficient (*R*_*s*_). For LINACS, the most suitable complexity metric was found to be the MU factor (Edge* R*_*s*_ = − 0.608, p < 0.01; TrueBeam *R*_*s*_ = − 0.739, p < 0.01). Regarding the specific breast cancer categories, the optimal complexity metrics were as follows: MAD (AUC = 0.917) for LBCS, MCS (AUC = 0.681) for RBCS, MU factor (AUC = 0.854) for LBCM and MAD (AUC = 0.731) for RBCM. On the Edge LINAC, the preferable method for breast cancers was MCS (left breast, AUC = 0.938; right breast, AUC = 0.813), while on the TrueBeam LINAC, it became MU factor (left breast, AUC = 0.950) and MCS (right breast, AUC = 0.806), respectively. Overall, there was no universally suitable complexity metric for all types of breast cancers. The choice of complexity metric depended on different cancer types, locations and treatment LINACs. Therefore, when utilizing complexity metrics to predict PSQA outcomes in IMRT for breast cancer, it was essential to select the appropriate metric based on the specific circumstances and characteristics of the treatment.

## Introduction

Intensity Modulated Radiation Therapy (IMRT), including Volumetric Modulated Arc Therapy (VMAT), was a common treatment technique for breast cancer radiotherapy^1^. Compared to 3D conformational radiotherapy (3D-CRT), IMRT could significantly improve target volume conformity and simultaneously spare normal tissue, resulting in reduced acute and late toxicities^2–4^. Due to multiple variable parameters, such as multi-leaf collimator (MLC) position, speeds, gantry rotation and beam stability, IMRT plan was very complex. However, more complex plans always had larger uncertainties in dose calculation and treatment delivery^5,6^. Therefore, evaluating treatment plans was an important step in the radiotherapy process that determined the characteristics of the plan selected for treatment^7^. While many benefits of radiation therapy in breast cancer treatment were well established, numerous challenges remained, particularly in achieving optimal target coverage while simultaneously avoiding organs at risk (OARs) in high-risk node-negative and node-positive breast cancer^1^. Some studies had found a linear no-threshold relationship between mean heart dose and the incidence of ischemic heart disease^8^. Therefore, our aim was to continually improve plan quality and treatment delivery in IMRT for breast cancer.

As we known, plan quality could be characterized by complexity metrics based on machine parameters and plan properties^9^. Depending on the sources of modulation, complexity metrics could generally be categorized into fluence map‐based metrics, and aperture‐based metrics^10^. Fluence map‐based metrics exclusively considered the resulting fluence from a modulated beam or plan. The hypothesis was that a highly heterogeneous fluence could reflect a highly level of complexity. However, these metrics were insensitive to the degeneracy of fluence maps^11^. For instance, the same fluence map could be the result of a single large beam or the sum of many small beams^12^. Several investigators have previously reported related studies^13–15^. For example, Llacer et al.^13^ designed the Fluence Map Complexity metric (FMC) to measure pixel values differences. Webb et al.^14^ introduced the modulation index (MI) to measure the variations between adjacent pixels differences. Coselmon et al.^15^ defined the similar metric, such as Plan Intensity Map (PIM). Aperture‐based metrics generally highlighted variations of the MLC positions during delivery^10^. The metric could be used to describe the variations in the mechanical and dosimetric parameters^11^. Numerous previous studies existed^10,12,16–19^. For instance, Du et al.^12^ defined the plan normalized Mus (PMU) to normalize the fraction dose. McNiven et al.^16^ provided the modulation complexity score (MCS) to evaluate the filed irregularity. Shen et al.^17^ reported the number of MU per CPs (MU/CP) to discuss the MLC motion. Masi et al. defined MCSv for VMAT. Some investigators also reported other metrics for the beam aperture position relative to the isocenter, such as the Cross-Axis Score (CAS)^19^, Mean Aperture Displacement (MAD)^10^.

The high degree of plan complexity could impact the accuracy of dose calculation and treatment delivery^20^. Early studied primarily focused on developing complexity metrics to predict the outcome of patient specific quality assurance (PSQA)^21,22^. Strategies were later proposed to streamline PSQA process and reduce workload^10,23^. Additionally, researcher had investigated the correlation between complexity metrics and plan quality^24^. Until now, most studies have concentrated on head and neck sites and pelvic cavity sites^25–28^, with limited studies conducted on breast cancers. Actually, breast radiotherapy presented unique challenges, such as the impact of regional node irradiation on survival and the need of cardiopulmonary sparing. Hence, the high quality and deliverable accurate treatment plan was urgently^1^. Short course regimen had become the major trend in adjuvant radiotherapy for breast cancer patients^29^. Our center has carried out several clinical trials on hypofractionated radiotherapy for breast cancer patients, including HARVEST (NCT03829553), ARROW (NCT04509648), SHIFT (NCT04926766). However, there have been no studies on the plan quality for these different trials, highlighting the importance of evaluating plan quality in this context. Various professional organizations strongly recommend PSQA^6,30,31^, which was considered the most accurate method for assessing delivery accuracy. Among the methods, portal dosimetry (PD) provided a reliable and convenient means of verifying the dose delivered to the patient^32–34^.

Generally, complexity was often associated with a large number of monitor unit (MU) and prescription (Gy)^18,35^. In this study, MU factor (MU/cGy) was selected as one of the complexity metrics due to its ease of use and effectiveness. Additionally, due to MCS incorporated fluence map and MLC-aperture complexity, the study also adopted the metric. Considering that breast cancer was located away from the central of body, the treatment plans for breast cancer often involved significant adjustments to the positions of the MLC apertures. The configuration of the MLC apertures could be an important factor for breast cancer treatment. Therefore, the study also selected other metrics to describe the displacement of the MLC apertures, such as CAS and MAD^10^. These metrics provided valuable insights into the status and impact of MLC aperture displacements in treatment plans for breast cancer.

In this study, our objective was to investigate the relationship between complexity and PSQA in IMRT for breast cancer. The ultimate goal was to enhance the efficiency and reliability of the IMRT treatment planning and delivery process for different kinds of breast cancers radiotherapy. By identifying these metrics, we could potentially improve the quality and effectiveness of treatment plans, leading to better outcomes for breast cancer patients.

## Materials and methods

### Treatment plans and dose verification

The dataset consisted of 80 breast cancer patients treated with IMRT at the Ruijin hospital, Shanghai Jiao Tong University School of Medicine during February 2019 to December 2022. This retrospective data collection was approved by our institutional review board (Shanghai Jiao Tong University Medical School Affiliated Ruijin Hospital) with waivers for the patient’s informed consent. Based on cancer location and type of breast surgery, the patient cohorts were divided into the following four categories: right-side breast cancer after breast conserving surgery (RBCS), right-side breast cancer after mastectomy (RBCM), left-side breast cancer after breast conserving surgery (LBCS), left-side breast cancer after mastectomy (LBCM), respectively. The prescription doses and fraction sizes used for breast cancer treatment in our center were as follows: 50 Gy/25 Fx, 40.05 Gy/15 Fx, 26 Gy/5 Fx, 50 Gy/25 Fx, depending on cancer location and type of breast surgery. Specifically, for RBCS and LBCS cases, we chose 40.05 Gy/15 Fx and 26 Gy/5 Fx, while For RBCM and LBCM cases, we selected 50 Gy/25 Fx.

Two LINACS (Edge and TrueBeam) (Varian Medical Systems, Palo Alto, CA, USA) were used to deliver 80 treatment plans of breast cancer IMRT. The MLC varied between the two machines. The Edge was configured with the HD120 MLC, renowned for its high-definition, offering enhanced precision and accuracy in radiation therapy treatments. On the other hand, the TrueBeam was equipped with a 120 MLC. The leaf width and mix area differed significantly. Edge encompassed a 22 cm × 40 cm field with 32 leaves at 0.25 cm width and the remaining at 0.5 cm. In contrast, the TrueBeam could generate a 40 cm × 40 cm field, featuring 40 leaves at 0.5 cm width and the rest at 1 cm. Among treatment plans, 42 were delivered by Edge, while the rest were done by TrueBeam. Table 1 detailed the distribution of plans investigated by treatment site. All treatment plans were designed by Eclipse treatment planning system (TPS, v.15.6), using the Analytic Anisotropic Algorithm TPS (AAA) for both LINACS, and 6 MV photon beams for all fields. All methodologies adhered strictly to relevant guidelines and regulations. The definition of target volume and OAR aligned with center protocols. Planning criteria stipulated that the target volume receiving the prescription dose must encompass over 95% the target volume. Additionally, dose constrains followed clinical trial protocols within our center (HARVEST (NCT03829553), ARROW (NCT04509648), SHIFT (NCT04926766)).

**Table 1:** Distribution of plans by treatment sites.

Treatment site	Prescription (Gy/Fx)	Edge (N = 42)	TrueBeam (N = 38)
RBCS	40.05 Gy/15 Fx	8	5
	26 Gy/5 Fx	8	4
LBCS	40.05 Gy/15 Fx	8	4
	26 Gy/5 Fx	7	6
RBCM	50 Gy/25 Fx	8	9
LBCM	50 Gy/25 Fx	3	10

*RBCS* right-side breast cancer after breast conserving surgery, *RBCM* right-side breast cancer after mastectomy, *LBCS* left-side breast cancer after breast conserving surgery, *LBCM* left-side breast cancer after mastectomy.

### Complexity metrics

Four previously established complexity metrics were adopted: MU factor, mean aperture displacement (MAD)^19^, cross axis score (CAS)^19^ and modulation complexity score (MCS)^16^. However, there were few studies that only focused on breast cancer radiotherapy. The detailed metrics, obtained by an in-house developed analysis tool, were described as follows:

1. MU factor, defined as the ratio of the total monitor units to the prescribed dose in cGy. Generally, increased complexity was often correlated with a large number of MU factor, and higher values of MU factor tended to result in lower passing rates, meaning that the plans were more complex. Hence, plans with higher MU factor were more complex^10^.
  $$MU\, factor=\frac{MU}{cG{y}_{plan}}$$
  where $MU$ was the $MU$ of plan and $cG{y}_{plan}$ was the prescription dose.
2. MAD, which described the displacement of the MLC apertures, was sensitive to the aperture per control point (CP) by considering the average of the distance between the center of the aperture distance between opposite leaf pairs and the MLC central axis^19,20^.
  $$MAD=\sum_{j=1}^{J}\frac{M{U}_{j}}{M{U}_{plan}}\sum_{i}^{I}\left(\sum_{k=1}^{K}\left|{m}_{k}\right|\right)\frac{M{U}_{i}}{M{U}_{beam}}$$
  where $J$ was the number of beams in the plan, $I$ was the number of segments in the beam, $K$ was the number of leaves in each opposing bank, and $m$ was the center of the aperture distance between opposing leaves.
3. CAS which described the displacement of across the central axis, was sensitive to the aperture per control point (CP) by considering the leaves that cross the MLC central axis^19,20^.
  $$CAS=\sum_{j=1}^{J}\frac{M{U}_{j}}{M{U}_{plan}}\sum_{i}^{I}\frac{N{\left(a>m\right)}_{i}}{N{\left(a>0\right)}_{i}}\frac{M{U}_{i}}{M{U}_{beam}}$$
  where $J$ was the number of beams in the plan, $I$ was the number of segments in the beam, $N$ was the number of leaf pairs not positioned under the jaws, $a$ was the aperture distance between opposing leaves and $m$ was the centre of the aperture distance between opposing leaves.
4. MCS incorporated the effects of fluence map complexity and MLC-aperture complexity, such as leaf positions, degree of irregularity in field shape, segment weight, and area. It was sensitive to the aperture area variability (AAV) and the leaf sequence variability (LSV) per segment for an IMRT beam. It indicated more complex plans with lower values^16,20^.
  $$MCS=\sum_{j=1}^{J}\left(\sum_{i=1}^{I}A{AV}_{{segment}_{i}}LS{V}_{{segment}_{\dot{i}}}\frac{M{U}_{{segment}_{\dot{i}}}}{M{U}_{beam}}\right)\frac{M{U}_{{beam}_{j}}}{M{U}_{plan}}$$
  where the aperture area variability (AAV) was used to characterize the variation in segment area relative to the maximum aperture defined by all of the segments and the leaf sequence variability (LSV) was defined to characterize the variability in segment shape for a specific plan. The definition of AAV and LSV was showed^16^.

### Pre-treatment dose verification measurement and evaluation

**Figure 1:**
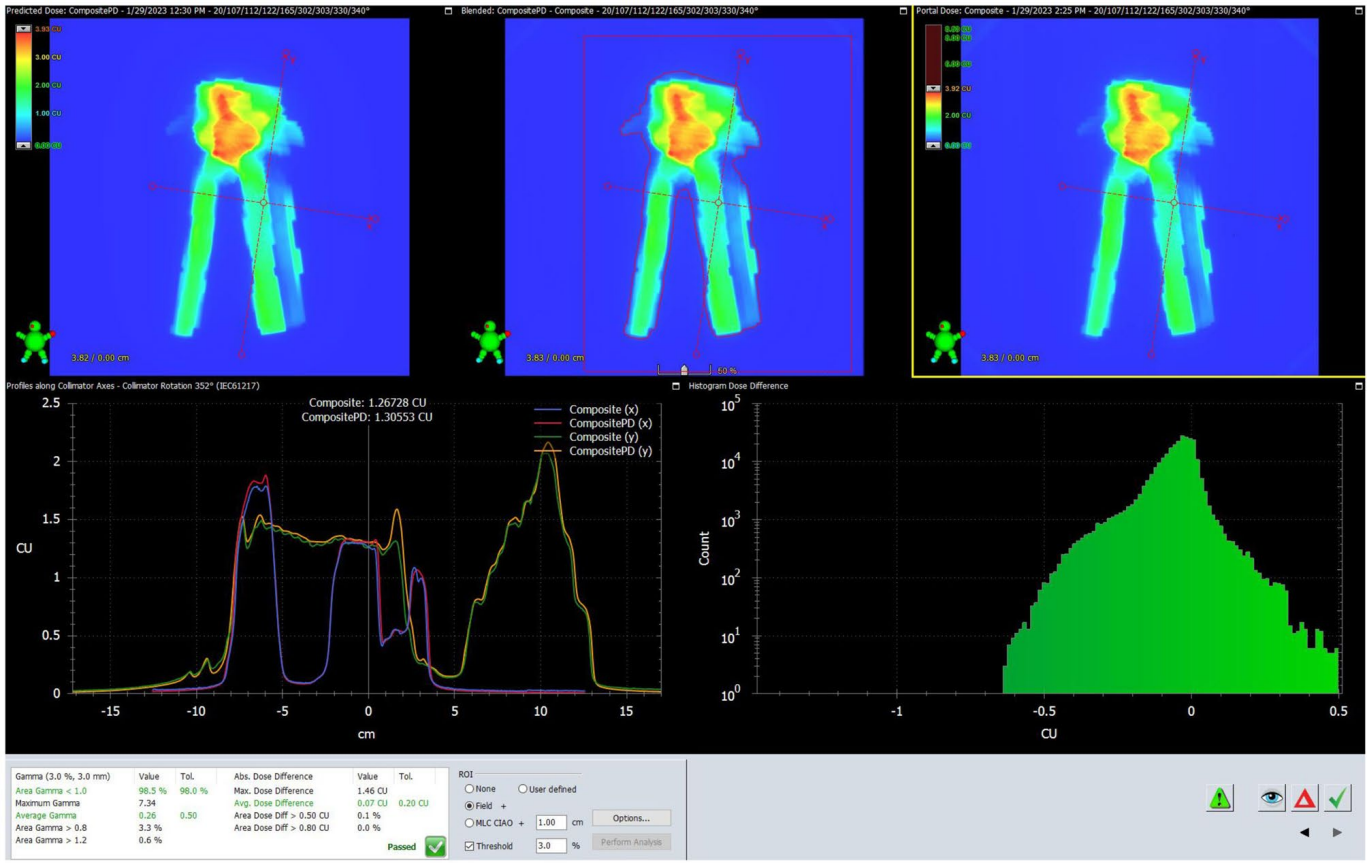
Portal dosimetry (PD) analysis interface.

Pre-treatment patient-specific quality assurance (PSQA) included dose verification and evaluation. Dose verification followed AAPM TG-218 guidelines^6^, using Varian Portal Dosimetry (V.10) for measurements, offering prompt setup, easy data acquisition and high spatial resolution^33^. The analysis was completed by comparing predicted dose images with measured images from the aSi imager on LINACS^19,20^. Before acquiring PD images, we calibrated the EPID image in two steps: beam profile correction and dose normalization using the calibration unit (CU)^36^. After measurement, we analyzed the results using integrated PD software. Plan evaluation employed gamma index (γ) criteria at 2%/2 mm and 3%/2 mm with a 10% low dose threshold and global dose normalization. Plans passed if 99% had γ (2%, 2 mm) < 1.0. Figure 1 showed the interface of PD analysis software, which consisted of predicted dose, blended dose and portal dose, profiles along collimator axes, histogram dose difference, respectively.

### Statistical analysis

AUC was a commonly used metric in various fields, particularly in the context of receiver operating characteristic (ROC) curves. Firstly, AUC was a robust metric for evaluating the classification performance. It provided a comprehensive assessment of the ability to distinguish between different classes, which was often crucial in predictive modeling tasks. secondly, AUC was threshold-independent, meaning it measured the model's performance across all possible classification thresholds. This was advantageous in scenarios where the optimal threshold might not be immediately clear or where different thresholds were of interest. Lastly, AUC facilitated comparisons between different models or variations of the same model. It allowed researchers to quantitatively compare the discriminatory power of different algorithms or feature sets. In this study, the use of AUC was used to evaluate the discriminative ability of complexity metrics for different breast cancer sites and LINACs. According to AUC values, we could easily check the classification performance. It provides a single, interpretable metric that summarizes the overall performance in distinguishing between the treatment plans of interest, ranging from 0 to 1. The higher the value, the better the performance. According to the recommendations^37^, a value that > 0.95 was considered near-perfect performance, > 0.9 was excellent, between 0.8–0.9 was good, 0.6–0.7 was fair, and 0.5–0.6 was regarded as poor performance. The distribution of each complexity metrics and gamma passing rate (GPR) was showed by scatter plots.

The correlation of gamma passing rate (GPR) and complexity metrics was examined, using Spearman’s rank correlation coefficient (*R*_*s*_). Strong correlation was indicated as |*R*_*s*_| ≥ 0.7, moderate as 0.7 > |*R*_*s*_| ≥ 0.5, weak as 0.5 > |*R*_*s*_| ≥ 0.3, and no correlation as 0.3 > |*R*_*s*_|. Statistical significance of a correlation was taken by a two‐tailed *p* value at *p* < 0.001.

## Results

### GPR

The distributions of the GPR for the TrueBeam and Edge LINACS were shown in Table 2, using the 3%/2 mm and 2%2 mm criteria, respectively. Because the means of GPR were greater than 98.5% and the result using the 2%/2 mm criteria was relatively lower, the study adopted the 2%/2 mm criteria. Firstly, the GPR of different suites from Edge was lower than that of the TrueBeam, except for LBCM. Meanwhile, the failed number was same for Edge and TrueBeam. Secondly, for the two LINACS, LBCS showed the more excellent than the RBCS at 40.05 Gy/15 Fx. For edge, LBCM was much higher than the RBCM, but it was opposite for TrueBeam. According to the same side comparative analysis, we found that RBCS, LBCS showed better performance, compared with RBCM, LBCM, respectively.

**Table 2:** The distributions of the GPR of the plans delivered on the Edge and TrueBeam LINACs.

	Prescription (Gy/Fx)	Edge (N = 42)		TrueBeam (N = 38)	
		3%/2 mm	2%/2 mm	3%/2 mm	2%/2 mm
Failed number		1	6	1	6
RBCS	40.05 Gy/15 Fx	99.81 ± 0.27	99.36 ± 0.61	99.94 ± 0.02	99.64 ± 0.15
	26 Gy/5 Fx	99.95 ± 0.03	99.80 ± 0.06	99.93 ± 0.03	99.83 ± 0.09
LBCS	40.05 Gy/15 Fx	99.89 ± 0.05	99.53 ± 0.16	100 ± 0	99.93 ± 0.05
	26 Gy/5 Fx	99.91 ± 0.03	99.91 ± 0.03	99.69 ± 0.11	99.97 ± 0.02
RBCM	50 Gy/25 Fx	99.53 ± 0.13	99.53 ± 0.13	98.71 ± 0.55	99.71 ± 0.09
LBCM	50 Gy/25 Fx	99.7 ± 0.17	99.3 ± 0.12	99.71 ± 0.05	99.17 ± 0.14

*Failed number* number of plans below tolerance.

### Complexity metrics

According to 2%/2 mm criteria, Table 3 showed the results of complexity metrics analysis for passing and failed plans. For Edge, the standard deviation (SD) of passing plans was smaller than the SD of failed plans. the values of MAD, CAS and MCS for means of passing plans were larger than the means of failed plans, but the value of MU factor was opposite. For TrueBeam, the agreements of SD were not obvious, the values of Mu factor, MAD and CAS for the means of passing plans were less than those for the failed plans, but, the MCS was bigger.

**Table 3:** Results of complexity metrics for the plans delivered on Edge and TrueBeam LINACs.

Complexity metric	Edge (N = 42)		TrueBeam (N = 38)	
	Mean of passing plans ± SD	Mean of failed plans ± SD	Mean of passing plans ± SD	Mean of failed plans ± SD
MU factor	5.2141 ± 2.9606	6.3333 ± 3.8217	6.6074 ± 3.1864	10.8114 ± 2.1818
MAD	45.3587 ± 8.9848	39.1745 ± 10.9257	48.0748 ± 11.2587	55.4114 ± 17.8605
CAS	0.9840 ± 0.0375	0.9489 ± 0.0667	0.9810 ± 0.0309	0.9962 ± 0.0054
MCS	0.1695 ± 0.0341	0.1502 ± 0.0478	0.1539 ± 0.0865	0.1136 ± 0.0542

*MU factor* monitor unit factor, *MAD* mean aperture displacement, *CAS* cross axis score, *MCS* modulation complexity score, *SD* standard deviation.

**Figure 2:**
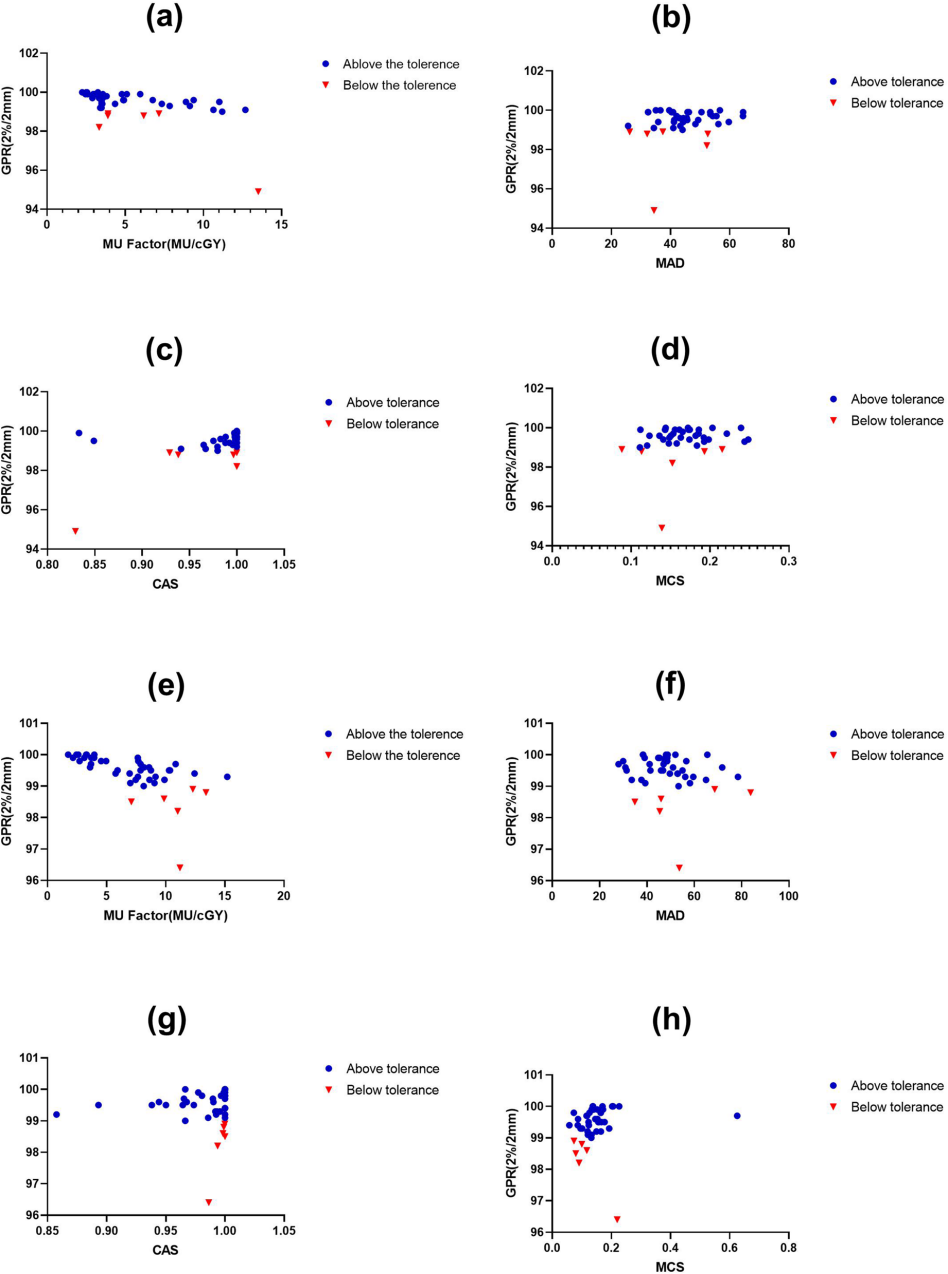
The scatter diagrams of complexity metrics and GPR for plans delivered on Edge and TrueBeam LINACs, using 2%/2 mm criterion. Plans with gamma passing rates above the 99% tolerance limit were marked as blue circles. and plans with gamma passing rates below the tolerance limit were marked as red triangles. (**a**–**d**) for Edge, the (**e**–**h**) for TrueBeam.

Figure 2 showed the scatter plots of the complexity metrics and quality assurance results. Comparing the same complexity metrics between the two LINACs, the distributions of MU, MAD and CAS were similar, except for MCS. For TrueBeam, the scatter plot of MCS exhibited smaller and more intensive variations. Additionally, the complexity values were similar, the variations of GPR were larger. Table 4 listed the corresponding values of the Spearman’s correlation coefficient with significance. For Edge, both the MU factor and CAS showed significant correlations, while the most meaningful metric for TrueBeam were the MU factor and MCS. However, MU factor exhibited a moderate correlation, particularly for TrueBeam LINAC.

**Table 4:** Correlations analysis of complexity metrics with GPR using 2%/2 mm criterion.

Complexity metric	Edge (N = 42, p value)	TrueBeam (N = 38, p value)
MU factor	− 0.608 (p < 0.01)	− 0.739 (p < 0.01)
MAD	0.173 (p < 0.273)	− 0.203 (p < 0.186)
CAS	0.506 (p < 0.01)	0.222 (p < 0.148)
MCS	0.149 (p < 0.347)	0.421 (p < 0.01)

### ROC curves

#### Different breast cancer sites

Table 5 listed AUC for each complexity metric about each breast cancer. LBCS obtained the highest value of complexity metrics except for the MU factor, while RBCS received the lowest value for complexity metrics except for the MU factor. Regarding MAD and MCS, the four different breast cancers exhibited the same order, with LBCS taking the top position, followed by RBCM, LBCM and RBCS.

**Table 5:** Areas under curve (AUC) of complexity metrics on different breast cancer sites.

Complexity metric	AUC for all LINACS			
	LBCS	RBCS	LBCM	RBCM
MU factor	0.625	0.620	0.854	0.577
MAD	0.917	0.680	0.729	0.731
CAS	1	0.5	0.813	0.692
MCS	0.896	0.681	0.708	0.712

#### Different LINACS

For different LINACS, complexity metrics were analyzed by ROC method. Table 6 displayed the AUC for left breast, right breast and whole breast for both Edge and TrueBeam, respectively. Concerning the left breast, the values of MAD, CAS and MCS for Edge were greater than those for TrueBeam. Regarding the right breast, the values of the MU factor and MCS for Edge followed the same pattern, while the values of MAD and CAS for Edge were smaller than those for TrueBeam. However, for the entire cancer sites, the pattern was opposite, with the values of the MU factor and MCS for Edge being smaller. Meanwhile, the values of MAD and CAS for Edge were greater than those for TrueBeam.

**Table 6:** AUC of complexity metrics on different LINACs.

Complexity metric	AUC for Edge			AUC for TrueBeam		
	Left breast	Right breast	All sites	Left breast	Right breast	All sites
MU factor	0.563	0.675	0.648	0.950	0.667	0.860
MAD	0.938	0.563	0.690	0.762	0.722	0.596
CAS	1	0.512	0.662	0.569	0.653	0.577
MCS	0.938	0.813	0.625	0.738	0.806	0.754

## Discussion

For 40.05 Gy/15 Fx, both LBCS and RBCS exhibited a similar trend for Edge and TrueBeam. For Edge, the GPRs of RBCS were smaller than the values of LBCS in both criterions of $\gamma$ (3%, 2 mm) and $\gamma$ (2%, 2 mm), Similarly, for TrueBeam, the GPRs of RBCS were also smaller in both criterions. However, the relationship varied for other prescriptions. For instance, the GPRs of RBCM was smaller than LBCM in $\gamma$ (3%, 2 mm), but the value was larger in $\gamma$ (2%, 2 mm). Additionally, it was observed that the GPRs of RBCS and LBCS were larger, compared to RBCM and LBCM. The difference could potentially be attributed to variations in the radiotherapy region, where the regions of the RBCS and LBCS were smaller than RBCM and LBCM. The study also noted that the SD of passing plans for Edge was larger, possibly due to Edge having a more sensitive GPR. This was attributed to the higher resolution MLC of Edge, making it often used for treatments with small field or more complicated targets^9^.

The means of passing and failed plans for complexity metrics showed different trends in two LINACS. For Edge, the mean of passing plans for MAD and CAS was larger, but for TrueBeam, the mean of failed plans for MAD and CAS was larger. This finding aligned with the previous research^11,20^, which indicated that the metrics (MAD, CAS) failed to show statistically significant correlation with the $\gamma$ passing rate. However, the trend was similar for MU factor and MCS on the two LINACS. For Edge and TrueBeam, the mean of passing plans for the MU factor was smaller than that of failed plans. but the result for MCS was opposite. This was consistent with the idea that the higher the value of GPR, the lower complexity, resulting in a smaller MU factor and the larger MCS.

The scatter plots further revealed the relationship between complexity metrics and GPRs of plans. the small ranges of values for failed plans were obvious observed in Fig. 2 (TrueBeam-CAS, TrueBeam-MCS). Among the failed plans, most MCS values were less than 0.15 and the CAS values were greater than 0.8. It was well-known that a complex plan means a larger uncertainty. The findings of Fig. 2 were in agreement with previous study^9^. Compared with CAS and MCS, the distributions of the MU factor and MAD were similar for the two LINACS.

Due to the highest correlation coefficient (− 0.608, − 0.739), the MU factor showed a moderate relationship for two LINACs. In addition, the values of 0.3 > |*R*_*s*_| showed that MAD had no correlation. other metrics had no consistent pattern. The result was similar with the former founding. Crow et al.^19^ reported that the metrics (MAD, CAS ) failed to show a statistically significant correlation with $\gamma$ passing rate for prostate cancer. According to |*R*_*s*_| (0.149, 0.421) for two LINACs, MCS did not show strong correlation. The finding was inconsistent with the former studies^19,38^.

The AUC of complexity metrics on different breast cancer sites showed different classification performance. Although the metrics (MAD, CAS and MCS) failed to show statistically significant correlation for whole breast cancer, they were significant for different breast cancer sites (LBCS, RBCS, LBCM and RBCM). In general, if AUC > 0.9, It represented excellent classification. Because the AUCs of the metrics (MAD, CAS and MCS) were nearly above 0.9 on LBCS, all of them showed good classification properties. For all complexity metrics, the ability of classification was weak on RBCS and RBCM. For left breast cancers, including LBCS and LBCM, Table 6 further displayed that the metrics (MAD, CAS and MCS) owned better performance on Edge, because the AUCs were above 0.9. It was consistent with ROC analysis of LBCS. Meanwhile, since the AUC was 0.95, the MU factor also showed excellent classification ability on the left breast cancer for TrueBeam.

Different from previous similar studies, the study exclusively focused on breast cancers. Additionally, it encompassed numerous factors, including breast cancers, fraction dose models, and treatment machines. The breast cancer was divided into LBCS, RBCS, LBCM, RBCM, left breast, right breast and whole breast. In contrast, The previous study typically considered the entire cancer and a single prescription, such as prostate cancer^39^. Comparing the whole breast cancers to their subdivisions, we observed variations in the results. For whole breast cancer, the MU factor exhibited the best classification ability, while MAD showed the worst classification performance. Compared with MAD, CAS, MCS, the MU factor was simple. In other word, it indicated that the simpler complexity metric yielded superior classification results. However, for LBCS and left breast cancer using Edge, the complexity metrics (MAD, CAS and MCS) served as excellent classification indices. For LBCM and left breast cancer using TrueBeam, the MU factor also proved to be a valuable metric. it further revealed that LBCS and left breast cancer were suitable application areas for complexity metrics. Although there was no universally applicable complexity metric for different breast cancers. Its suitability varies with different cancer type, location and LINAC treatment. The study revealed that MAD, CAS and MCS were excellent classification indices for LBCS, with no universal law found for different breast cancer sites and complexity metrics. Additionally, the study analyzed the distribution features of passing and failed plans for complexity metrics, revealing different patterns for the two LINCAs. All of the above findings held clinical significance. Specifically, the study contributed to a deeper understanding of complexity metrics for different breast cancers. It highlighted that not all complexity metrics (MU factor, MAD, CAS and MCS) was universally useful for different breast cancers. And it shed light on the distribution patterns and application ranges of these complexity metrics for different breast cancers. At times, it could potentially impact treatment decisions. For example, based on the values of MAD, CAS or MCS, we could determine to whether PSQA for the plan and design new plan or not. Furthermore, these values also affected plan quality. We believed that the study contributed to the understanding of the relationship between complexity metrics and PSQA for breast cancer. It could assist clinicians and medical physicists in reducing PSQA time and improving the efficiency.

Of course, there were some challenges or limitation. For example, PD was a valuable tool in radiation therapy, but like any technique, it had its limitations. Firstly, PD relied on beam model to calculate the expected portal dose images. Any discrepancies in the model could lead to errors in the dose calculation. Therefore, before each patient dose verification, dose calibration for LINACS and EPID image should be carried out. Secondly, PD was sensitive to patient anatomy changes. It might not account for daily anatomical variations in the patient, such as changes in organ position and shape. This could lead to discrepancies between the planned and delivered doses, particularly in cases of significant anatomical changes. It was a common problem for offline dose verification. Thirdly, PD only provided 2D information of the dose distribution, which might not capture the full complexity of three-dimensional dose variations within the patient. In the future, we would plan to compare 2D and 3D dose distribution, using multiple dose verification tools. Additionally, ROC was only a method of analysis, and AUC did not fully represent the performance of complexity metrics. The result of the study should be verified by enough samples, including all kinds of breast cancer sites and many different types of plans (IMRT, VMAT). Although we used many methods to reduce errors, some were unavoidable. In the future, we would adopt enough samples for verification and expand applied range to other cancers.

## Conclusion

The relationship between four complexity metrics (MU factor, MCS, MAD, CAS) and PSQA in IMRT for breast cancer was evaluated. We found that there was no universally suitable complexity metric for different breast cancers. It varied with different types, locations and treatment LINACs. Concerning treatment LINACs, the most appropriate complexity metric was the MU factor, and for LBCS, RBCS, LBCM and RBCM, the best indices were MAD, MCS, MU factor and MAD, respectively. Additionally, the most suitable method for left and right breast IMRT was MCS on Edge LINAC, and it became MU factor and MCS on TrueBeam LINAC, respectively. In conclusion, when using complexity metrics to predict PSQA outcomes in IMRT for breast cancer, we should choose the suitable complexity metric based on the types, locations and treatment LINACs.

## Data Availability

The datasets used and/or analysed during the current study available from the corresponding author on reasonable request.
